# Identification of regulatory network topological units coordinating the genome-wide transcriptional response to glucose in *Escherichia coli*

**DOI:** 10.1186/1471-2180-7-53

**Published:** 2007-06-08

**Authors:** Rosa María Gutierrez-Ríos, Julio A Freyre-Gonzalez, Osbaldo Resendis, Julio Collado-Vides, Milton Saier, Guillermo Gosset

**Affiliations:** 1 Departamentos de Microbiología Molecular, Instituto de Biotecnología, Universidad Nacional Autónoma de México, Apdo. Postal 510-3, Cuernavaca, Morelos 62250, México; 2 Ingeniería Celular y Biocatálisis, Instituto de Biotecnología, Universidad Nacional Autónoma de México, Apdo. Postal 510-3, Cuernavaca, Morelos 62250, México; 3 Programa de Genómica Computacional, Centro de Ciencias Genómicas, Universidad Nacional Autónoma de México; 4 Division of Biological Sciences, University of California at San Diego, La Jolla, CA, 92093-0116, USA

## Abstract

**Background:**

Glucose is the preferred carbon and energy source for *Escherichia coli*. A complex regulatory network coordinates gene expression, transport and enzyme activities in response to the presence of this sugar. To determine the extent of the cellular response to glucose, we applied an approach combining global transcriptome and regulatory network analyses.

**Results:**

Transcriptome data from isogenic wild type and *crp*^- ^strains grown in Luria-Bertani medium (LB) or LB + 4 g/L glucose (LB+G) were analyzed to identify differentially transcribed genes. We detected 180 and 200 genes displaying increased and reduced relative transcript levels in the presence of glucose, respectively. The observed expression pattern in LB was consistent with a gluconeogenic metabolic state including active transport and interconversion of small molecules and macromolecules, induction of protease-encoding genes and a partial heat shock response. In LB+G, catabolic repression was detected for transport and metabolic interconversion activities. We also detected an increased capacity for *de novo *synthesis of nucleotides, amino acids and proteins. Cluster analysis of a subset of genes revealed that CRP mediates catabolite repression for most of the genes displaying reduced transcript levels in LB+G, whereas Fis participates in the upregulation of genes under this condition. An analysis of the regulatory network, in terms of topological functional units, revealed 8 interconnected modules which again exposed the importance of Fis and CRP as directly responsible for the coordinated response of the cell. This effect was also seen with other not extensively connected transcription factors such as FruR and PdhR, which showed a consistent response considering media composition.

**Conclusion:**

This work allowed the identification of eight interconnected regulatory network modules that includes CRP, Fis and other transcriptional factors that respond directly or indirectly to the presence of glucose. In most cases, each of these modules includes genes encoding physiologically related functions, thus indicating a connection between regulatory network topology and related cellular functions involved in nutrient sensing and metabolism.

## Background

In their natural environments, bacteria must adapt to changing physicochemical conditions. Adaptation responses are controlled by a complex network of sensory and regulatory proteins that modulate cellular functions at the transcriptional and posttranscriptional levels. Nutrient availability, ranging from sufficiency to total deprivation, is one of the environmental variables the cell is constantly sensing. Among nutrients, carbohydrates are particularly important to the cell since they are utilized as both carbon and energy sources. Glucose is the most abundant aldose in nature, being present mostly in polymeric states as starch and cellulose [1]. This sugar is the preferred carbon and energy source for the gram-negative bacterium *Escherichia coli *(*E. coli*) [2]. Specialized protein systems are present in *E. coli *to sense, select and transport glucose. This sugar is internalized and phosphorylated by the phosphoenolpyruvate:sugar phosphotransferase system (PTS). This system catalyzes group translocation, a process that couples transport of sugars to their phsosphorylation. The PTS is widespread in bacteria but absent in Archaea and eukaryotic organisms [3,4]. It is composed of soluble non sugar-specific protein components, Enzyme I (EI) and the phosphohistidine carrier protein (HPr) which relay a phosphoryl group from the glycolytic intermediate, phosphoenolpyruvate (PEP), to any of the different sugar-specific enzyme II complexes. Glucose is imported by the II^Glc ^complex, composed of the soluble IIA^Glc ^enzyme and the integral membrane permease IICB^Glc ^[5].

The preferred nutritional status of glucose for *E. coli *is evidenced by the observed repression and inhibition exerted by this sugar on gene expression and the activities of enzymes and transporters related to the consumption of other carbon sources. This example of global regulation is called carbon catabolite repression (CCR) [2]. As a sensor of the presence of glucose in the external medium, the PTS plays a central role in CCR. When glucose is present in the medium and it is being transported by the PTS, the IIA^Glc ^protein is non-phosphorylated, and in this state, it binds to various non-PTS permeases inhibiting uptake of other carbon sources. This form of IIA^Glc ^also binds to the enzyme glycerol kinase (GK), inhibiting its activity. When glucose is absent from the culture medium, IIA^Glc ^is mainly in its phosphorylated state. In this condition, IIA^Glc^~P binds to the enzyme adenylate cyclase (AC), activating its cyclic AMP (cAMP) biosynthetic capacity. Therefore, cAMP concentrations increase in the cell. Then cAMP binds to the cAMP receptor protein (CRP) and promotes the induction of catabolite-repressed genes[2].

The global transcriptional response of *E. coli *to different nutrient/environmental conditions has been studied using microarray technology. These studies have revealed complex genome-wide expression patterns that reflect the roles of different cellular regulators on cell adaptability and survival. Some of these works have focused on analyzing the effects on global transcription patterns of growing *E. coli *in minimal or complex media with different glucose concentrations [6-9]. These studies have enabled the identification of genes whose transcript levels change in response to each specific condition. In order to characterize the cellular response to glucose, conditions must be chosen that represent sufficiency and the complete lack of this nutrient. A comparison of genome-wide transcriptome patterns between strains grown under these conditions should be adequate for identifying the group of genes displaying a transcriptional response to glucose which we term, the "glucose stimulon". In this work, we use transcriptome data, collected under conditions of glucose absence or excess in a complex medium. Analyses of the data set were used to identify the genes encoding cellular functions that respond to this stimulus and enable the cell to adapt to nutrient availability. Topological analysis of the regulatory network involved in this response revealed modular organization where global and local transcriptional factors integrate different signals to detect and respond to the presence of glucose.

## Results and Discussion

### Global transcriptome response to the presence of glucose in complex medium

Transcriptome data was obtained from previously reported experiments performed with *E. coli *strain BW25113 and an isogenic *crp *mutant (LJ3017)[10]. These strains were grown in LB medium with (LB+G) or without (LB) 0.4% glucose. Total RNA was extracted from each condition, processed and hybrydized to the Affymetrix *E. coli *array which includes 4327 genes [11]. Three data sets were obtained for each of three experimental conditions: wild type grown in LB medium (WT), wild type grown in LB medium + glucose (WTg) and a *crp*^- ^mutant grown in LB medium (CRP). Starting with these data, differentially transcribed genes were selected using an outlier iteration method [12-14]. Analysis of the data from the WTg/WT log ratios, allowed the identification of genes having a significant change in transcript level (Table 1) [15]. 180 genes showed increased and 200 reduced relative transcript levels. Of these 380 genes, 87 belong to the hypothetical, unknown class.

**Figure 1:**
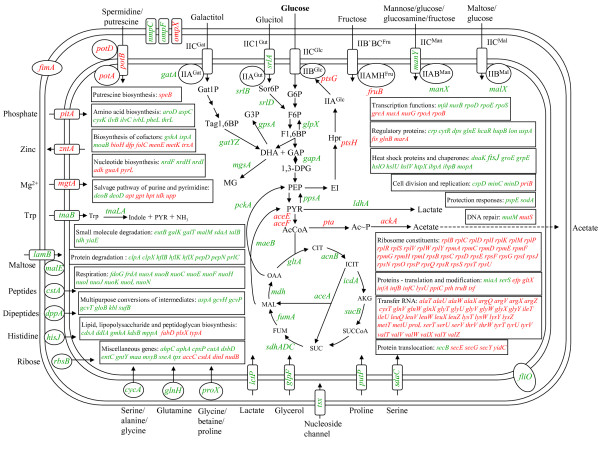
**Transcriptome profile of *E. coli *comparing growth in LB+G to that in LB. Genes displaying higher and lower transcript levels due to the presence of glucose are shown in red and green, respectively**. Abbreviations: 1,3-DPG, 1,3-diphosphateglycerate; AcCoA, acetyl coenzyme-A; Ac~P, acetyl phosphate; AKG, á-ketoglutarate; CIT, citrate; DHA, dihydroxy-acetone-phosphate; F1,6BP, fructose-1,6-bisphosphate; F6P, fructose-6-phosphate; FUM, fumarate; G3P, glycerol-3-phosphate; G6P, glucose-6-phosphate; GAP, glyceraldehyde-3-phosphate; GAT1P, galactitol-1-phosphate; ICIT, isocitrate; MAL, malate; MG, methylglyoxal; OAA, oxaloacetate; PEP, phosphoenolpyruvate; PYR, pyruvate; Sor6P, sorbitol-6-phosphate; SUC, succinate; SUCCoA, succinyl-CoA; Tag1,6BP, tagatose-1,6-bisphosphate; Trp, L-tryptophan.

Figure 1 shows an integrated view of the transcriptional responses and their roles in cell physiology of the 293 genes having a known function and found to respond to glucose. As can be seen, the presence of glucose induced the expression of genes encoding the general PTS protein Hpr and the glucose-specific IICB^Glc ^permease. This response is expected to increases cellular glucose transport capacity [16]. Glucose-dependent induction was also detected for genes encoding proteins involved in the import of polyamines, inorganic phosphate and magnesium ions, thus suggesting that these nutrients are required to sustain the higher growth rate observed in the LB+G medium. In contrast, glucose had a repressive effect on genes encoding transporters and periplasmic receptor proteins related to the import of alternative carbon and carbon-nitrogen sources. These included: amino acids, carbohydrates, lactate, glycerol, peptides, dipeptides and nucleosides. Furthermore, a reduction in transcript levels was observed for genes encoding proteins involved in the catabolism of several sugars and amino acids. This transcriptome pattern is the expected result of carbon catabolite repression exerted by glucose [2].

The presence of glucose had a significant effect on transcript levels of genes encoding enzymes of central metabolism. Upregulation with glucose was detected for the genes encoding the E1 and the lipoate acetyltransferase/dihydrolipoamide acetyltransferase subunits of the pyruvate dehydrogenase multienzyme complex (Pdh) as well as the genes encoding phosphotransacetylase and acetate kinase, that constitute an acetate synthesis pathway. On the other hand, downregulation was observed for genes encoding nearly all enzymes involved in gluconeogenesis, the TCA cycle and the glyoxylate bypass[17]. The observed responses are consistent with the expected glycolytic metabolism induced by exogenous glucose.

Functions related to nucleotide biosynthesis and salvage pathways of purines and pyrimidines were found to change in response to glucose. Growth in LB+G medium reduced transcript levels of genes encoding proteins involved in (deoxy)ribose phosphate degradation, as well as the salvage pathways for both adenine, hypoxanthine, and their nucleosides and pyrimidine ribonucleotides and pyrimidine deoxyribonucleotides. By contrast, transcript levels for genes encoding enzymes that participate in the *de novo *biosynthesis of purine and pyrimidine ribonucleotides were increased. These results suggest that the cell exists in a metabolic state where it is importing and interconverting ribo and deoxyribonucleotides present in the LB medium, but addition of glucose induces another state where *de novo *synthesis capacity is increased.

For genes encoding enzymes of amino acid metabolism, different effects of glucose were observed. Downregulation in LB+G medium relative to LB medium was detected for genes involved in biosynthetic pathways for aromatic amino acids, aspartate, cysteine, isoleucine-valine, phenylalanine and threonine. Interestingly, downregulation was also observed for genes encoding activities involved in the degradation of aspartate, cysteine, glycine and threonine. In addition, as mentioned above, a decrease in transcript level was detected for genes encoding importers for alanine, glutamine, glycine, histidine, proline and serine. These results indicate a reduction in import and degradation capacity for several amino acids when growing in LB+G medium. This can be explained considering that in this condition amino acids utilization as carbon sources should be significantly reduced. The apparent reduction in the demand for external amino acids to be used as alternative carbon sources or building blocks could also be a consequence of increased capacity for the *de novo *synthesis of amino acids once glucose is available. However, as noted above, induction of genes of amino acid synthesis pathways was never observed, and, in fact, repression was observed for several pathways. Therefore, the effects of glucose on degradative and biosynthetic capacities do not seem to be global but amino acid-specific.

A general trend of upregulation in LB medium was detected for genes encoding proteases, indicating higher proteolytic activity under this condition when compared to growth in LB+G medium. This makes sense since peptide degradation and protein turnover can provide carbon and energy for biosynthetic purposes in the absence of glucose. A similar pattern was observed for heat shock proteins and chaperones. These results suggest a higher protein turnover rate in the absence of glucose. The possible presence of partially degraded or misfolded proteins when the cells are growing in LB medium could cause the induction of heat shock proteins and chaperones, as has been previously reported [18,19]. It should be pointed out that several of the induced proteases are involved in regulatory processes (Fig. 1). The overall regulatory effects of such a response remain to be determined. A decrease in transcript level for heat shock proteins and chaperones upon growth in LB+G medium indicates that functions related to protein turnover are reduced by the presence of glucose, suggesting a lower capacity or need to use of amino acids derived from proteins as sources of carbon or protein constituents, or that proteins are more stable in an energized cell.

Medium composition had an important effect on genes encoding proteins involved in translation. Increased transcript levels were observed for genes encoding 20 of the 30 ribosomal protein components of the 50S subunit and 16 of the 22 ribosomal proteins of the 30S subunit. Also increased were transcript levels for 46 tRNA genes, grouped in 14 transcriptional units (TUs). Two of these TUs include genes *rrnA *and *rrnD*, encoding two of the seven 16S ribosomal RNAs. These genes are known to be subject to growth rate-dependent regulation [20]. In cultures used to obtain the RNA to generate the transcriptome data, a 5% higher growth rate was observed when comparing LB+G to LB conditions [10,21]. Therefore, induction of genes encoding ribosomal proteins, tRNAs and rRNAs is an expected response to the higher growth rate in LB+G medium.

Cell division and replication functions were found to respond to medium composition. Glucose lowered transcript levels for genes encoding DNA replication inhibitor protein CspD and the cell division inhibitor and membrane ATPase MinCD of the MinC-MinD-MinE and DicB-MinC systems. The *cspD *gene is known to be induced upon glucose starvation [22,23]. An increase was observed in transcript level for the gene encoding PriB protein that is a component of the multiprotein complex called primosome. This complex is believed to be involved in the restart of stalled DNA replication forks. The concerted down regulation of inhibiting and up regulation of activating chromosomal replication and cell division functions is consistent with a cellular response to favorable growth conditions afforded by the presence of glucose.

We found several transcription-related functions to be induced by glucose, these included the α and β subunits of the RNA polymerase core enzyme, as well as the elongation and antitermination factors GreA, NusA and NusG. Under the same growth condition, repression was observed for genes encoding the transcriptional termination factors NusB and Mfd. Thus, the observed responses for these functions are consistent with an expected increase in the transcriptional rate and efficiency caused by the increased biosynthetic demand of the higher growth rate in the presence of glucose. However, we also detected a reduction in transcript levels for genes encoding sigma 70, sigma E and sigma 38. It remains to be determined what the net consequences on the transcriptional capacity and RNA polymerase promoter selectivity would be, resulting from the observed expression changes.

Increased transcript levels were detected for the gene encoding agmatine ureohydrolase (*speB*), an enzyme involved in the putrescine biosynthetic pathway. Genes encoding the integral membrane component of the flagellar export apparatus FliO (*fliO*) displayed a decrease in transcript levels. Putrescine synthesis in *E. coli *can proceed from the decarboxylation of arginine to agmatine and its subsequent hydrolysis to putrescine, reactions catalyzed by the products of genes *speA *and *speB*, respectively[24]. The higher transcript levels when growing in LB+G medium for *potABD *and *speB *encoding components of the spermidine/putrescine ATP-dependent importer and an enzyme of the putrescine biosynthetic pathway, respectively, are indicative of an increased demand for polyamines when conditions favor a higher growth rate for *E. coli*. Growth in medium containing glucose is known to repress flagellum synthesis[25]. Gene *fliO *is a member of the flagellar class II operon *fliLMNOPQR*, encoding proteins of the export apparatus and the motor/switch complex for flagellar function. This operon can be transcribed by either sigma 70 or the flagellum-specific sigma 28[26].

This analysis enabled us to demonstrate that glucose causes a change in transcript levels of 380 genes, grouped in 142 TUs, corresponding to 9% of the *E. coli *genome. If it is assumed that complete operons are induced when at least one gene member is detected in the microarray, then, this number would increase to 492 genes, corresponding to 11% of the *E. coli *genome. The comparison of the observed transcriptome pattern under the two nutritional conditions studied revealed global responses that involve functions not limited to nutrition/metabolism. Although *E. coli *displays high and similar growth rates in LB and LB+G media, this analysis reveals different transcriptome patterns that are consistent with distinct physiological states under these two conditions.

### Transcriptional regulatory elements and mechanisms involved in glucose responses in *E. coli*

In recent years, many groups have concentrated on the study of the transcriptional responses of genes that integrate the regulatory network (RN) of some model organisms such as *S. cerevisiae *and *E. coli *[27,28]. Some of these studies have analyzed the connectivities between the genes and TFs to understand topological properties of the RN [28,29] and infer modules that reflect a correlation between physiological and genetic responses. External stimuli provoke changes in the RN that help the cell to contend with a changing environment. The development of microarray technologies, gives us the opportunity to study globally the expression of genes in response to a given stimulus and try to detect the part of the RN (subnetwork) responsible for the adaptative response.

The second part of this study consisted on the identification of the transcriptional RN involved in the observed glucose responses. This analysis represents an approach to understand at a systems level the behavior of the RN. The complete RN in the current version of the RegulonDB data base [30] represents 693 interactions involving 402 genes and 89 TFs. From the 380 regulated genes identified in the WTg/WT experiment, 142 possess a known regulatory interaction in RegulonDB. For these genes, we extracted from RegulonDB, the known information about TFs involved in their regulation. With this information, the RN was defined. We organized the regulatory interactions (RI) in strict simple and complex regulons (as previously described [31]. This data organization enabled us to analyze the interplay of the TFs involved in the regulatory changes of expression shown in the microarray data. We observed that 114 of these genes are regulated or coregulated by a global TF [32] (CRP, FNR, IHF, Fis, ArcA, NarL or Lrp), and only 28 of them don't interact with a global regulator (*zntA, mtgA, mgrB, metK*, *sufB, lon*, *cysK, uspA, fliO, fruB, pps, pckA, entC, nrdF, nrdH, nrdI, gatY, gatZ, gatA, ilvC, rpoD, rpsU, ahpC, hisJ, sufB, glnB, speB, proX*). The TFs involved in the regulation of these 28 genes are GadX, CysB, FadR, FhlD, FruR, Fur, GatR, LexA, OxyR, IlvY, MetJ, PhoB, PurR and NtrC.

Our data revealed a very small number of genes encoding TFs (*hupB*, *crp, fis, marA, cytR, yagA *and *hcaR*) that responded to the conditions studied (presence of glucose or loss of *crp *function). Although this will be explained in detail below, it should be pointed out that several of these TFs are involved in the regulation of a large number of the genes displaying a significant response to glucose.

As previously reported, we found that glucose responses are highly dependent on the TF, CRP [2], which is a global dual regulator, that governs the expression of at least 140 genes and corregulates gene expression with 75 other TFs [2]. In *E. coli*, CCR is mainly mediated by the PTS. When glucose is present in the culture medium, protein IIA^Glc ^lacks the capacity to activate adenylate cyclase; therefore, cAMP is present at relatively low levels. Lacking cAMP, the CRP protein cannot bind DNA and activate catabolite-repressed genes [3]. Therefore, in the presence of glucose, CRP is unable to exert its usually positive effect on its regulated genes. The microarray and RegulonDB data revealed that of the 142 genes with known regulatory interactions, 50 are CRP regulated. Seven of these genes (*crp*, *cstA*, *ivbL*, *ilvB*, *putP*, *spf *and *trxA*), are regulated only by CRP. The other 42 genes are corregulated by CRP and one or more of 26 other TFs. From the 50 CRP affected genes, RegulonDB data indicates that 34 of them are activated by CRP and other TFs, 7 of them are exclusively activated by CRP, 6 are dual regulated and 3 genes present two CRP sites with opposite functions (Table 1). Except for the gene *putP *the seven genes that are solely regulated by a negative CRP binding site are induced in our experiment as expected. In the cases of *truB*, *infB nusA *and *rpsO*, the effect of Fis seems to enhance the expression of these genes, suggesting that the repression of *putB *could occur because of the presence of another TF, alternative regulatory mechanisms or additional CRP binding sites acting as positive regulators.

Transcriptome data showed that some of the genes positively regulated by CRP were down-regulated, in spite of the presence of other positive TFs like MalT, TorR and FNR. This effect had been previously described for the *melAB *and *malM *promoters [33,34], where CRP acts as a coactivator with a second TF. In our data, we found this response for the *malE *and *malM *genes, in which CRP triggers the repositioning of MalT to an appropriate activating position, causing the genes to be expressed [34]. The rest of the CRP regulated genes that do not appear repressed by glucose, are exclusively negatively regulated by CRP (*trxA*), or have one or more regulators that may counteract the effect of CRP (Table 1).

We found an important number of genes to be under the influence of Fis. RegulonDB reports 94 genes regulated by Fis. Our RN data showed 52 genes affected in the presence of glucose by Fis, grouped in 21 transcription units, out of which 48% belong to the Fis simple regulon, sharing some interesting characteristics: a) All are positively regulated by Fis, b) all are tRNA genes and c) when a binding site was reported, the central position varies between -66 and -75. Other members of the group, like *tyrT*, *alaT *and *tyrV *share the same characteristics except that they have three or two Fis binding sites. In the case of the genes *alaU*, *ileU *and *thrV*, a site for the nucleoid-structuring protein (HNS) has been characterized. It has been reported that the Fis site located near the promoter (between -71 and -78) is essential for promoter activation [35]. We observed another group of Fis-regulated genes that share their regulatory region with accessory TFs and additional Fis sites. The group of genes including *truB*, *b3170*, *nusA*, *infB *and *rpsO*, are co-transcribed by the complex regulon – ArgR(-), CRP(-) and Fis(+) --. According to our data, this group appeared coordinately induced. We assume that this induction is caused by Fis activation together with no repressing effect of CRP (inactive in the presence of glucose) or ArgR.

The *nuo *genes, encoding the proton-translocating NADH:quinone oxidoreductase, appeared coordinately expressed, and all of the *nuo *genes are organized in a 13 genes operon (one of the longest transcription units in the genome). It has been reported that regulation of the expression of the *nuo *operon is subject to ArcA, that mediates anaerobic repression and NarL that mediates anaerobic activation in the presence of nitrate [36]. FNR and IHF act as weak repressors under anaerobic conditions [36], and Fis has been reported to stimulate expression of the operon in early exponential phase and to a lesser extent in the late exponential and stationary growth phases [37]. No significant difference in dissolved oxygen tension is expected when comparing cultures in LB or LB+G. Therefore, it can be speculated that transcriptional downregulation of the *nuo *operon is caused by medium composition or cell growth rate by an unknown mechanism. We detected an increase in the activity of *marA*, a gene that codes for the MarA TF, which is known to regulate its own expression [36]. Previous reports demonstrated that Fis stimulates expression of *marA *when MarA acts as an activator [38].

CRP has been described as the master regulator largely responsible for the expression pattern when *E. coli *is grown in glucose as the carbon source. However, very little is known about the influence of Fis on the gene expression pattern under the same conditions. We found a previous report showing that Fis is the factor mostly responsible for catabolite repression at the *nrf *promoter [39]. Experiments from other groups revealed that Fis assists both Mlc repression and CRP-cAMP activation of *ptsG *through the formation of Fis-CRP-Mlc or Fis-CRP nucleoprotein complexes at the *ptsG *promoter depending on the glucose availability in the growth medium [39,40]. Considering the large fraction of genes regulated by Fis identified in our study, it is clear that this TF has an important role in the cellular response to glucose.

### Cluster analysis of transcriptome data for selected genes of wild type and *crp*^- ^strains

**Figure 2:**
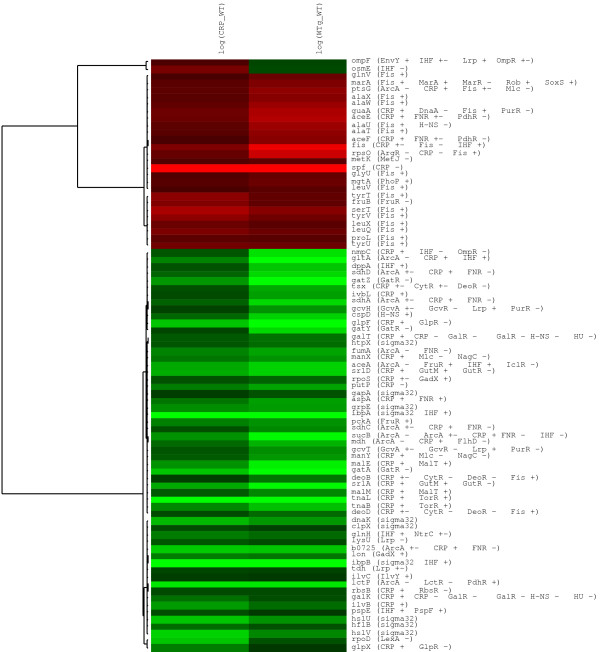
**Cluster analysis of genes responding to both the presence of glucose and the loss of CRP**. Red color indicates the induction state of the genes, and green color represents the repressed state. The name of each gene and the transcriptional factors involved in its regulation are indicated on the right side.

It has been proposed that most of the genes affected by the presence of glucose are directly or indirectly modulated by CRP. Glucose has an inactivating effect on CRP activity mainly by virtue of depressing cAMP levels. An analysis that compares transcriptome patterns between a *crp*^- ^mutant and the isogenic wild type strain grown in the presence of glucose could give clues about what genes are differentially expressed under these conditions. The results obtained from such analyses should identify which genes have a CRP-dependent response to glucose. To help in identifying the role of CRP in the response to glucose, in this analysis we included transcriptome data from a *crp *mutant strain grown in LB conditions. A subset of 83 genes that displayed a significant response to both WTg/WT and *crp*/WT conditions was used in this analysis. For this purpose, using the results of microarrays conducted under these two previous conditions, we used a hierarchical clustering algorithm to evaluate the behavior of the genes shared under both conditions [41]. Figure 2, presents the clustering results, including labels with gene names and the corresponding regulating TFs. The cluster results showed that nearly all genes present the same response under both conditions. This indicates that the observed transcriptional response is dependent on CRP; however, it is not possible to determine from these results if the effect is direct or indirect. From this group of 83 genes, 25 displayed higher transcript level in the presence of glucose, with 13 being regulated solely by Fis, and 9 by this and other TFs. Among the latter group is the gene *fis*, regulated by CRP, Fis and IHF. It is noteworthy that under both conditions, the genes up-regulated by Fis, including *fis*, are significantly induced, suggesting that CRP plays an important role in the regulation of this gene. This result indicates that CRP is acting as a repressor of *fis *transcription. It has been reported that CRP together with Fis represses *fis *transcription during the exponential growth phase[42]

The TU including genes *aceE *and *aceF *is positively regulated by CRP, dualy regulated by FNR and negatively regulated by PdhR. Considering that upregulation was also observed in the *crp *mutant, it can be inferred that CRP is not participating in this response. No changes in dissolved oxygen tension are expected when comparing cultures in LB or LB+G; therefore regulation by FNR can be ruled out. On the other hand, in LB+G, glucose catabolism should cause an increase in pyruvate concentration when compared to growth in LB medium. If this is the case, pyruvate can bind to and inactivate the repressor PdhR, thus causing the observed induction.

Another remarkable observation resulted from examination of the genes that appeared repressed, but a binding site for CRP or for other TFs regulated by CRP has not been identified (considering the information available in Regulon DB or EcoCyc). This was the case for the *pckA*, *lon*, *gatA*, *gatZ*, *gatY*, *gcvH*, *gcvT, osmE*, *dppA*, *pspE*, *ilvC*, *rpoD*, *lysU*, and *tdh *genes. Some of them, as mentioned before, are carrier proteins related to the import of alternative carbon and nitrogen sources (*gatA*, *gatZ*, *gatY *and *dppA*). The genes *aceA *and *pckA *deserve special attention because their regulator, the fructose repressor (FruR), is known to be partially inactivated in the presence of glucose. Fructose-1-phosphate and fructose-1-6-diphosphate, (direct products of glycolysis), bind to FruR and inactivate its DNA-binding capacity [41,43]. As FruR positively regulates the expression of these two genes, the inactivation of the regulator causes the gene to be down regulated, a result that can be observed in our data. In addition, we found the gene *fruB *to be upregulated by the presence of glucose. This gene is repressed by FruR. In this case, we again find evidence of FruR inactivation by glycolytic intermediates[44]. These are significant results, as they allowed us to infer that a higher internal level of the glycolytic intermediate fructose-1-6-bisphosphate is present in the cells growing in the LB+G medium, when compared to the LB grown cells.

The genes *osmE *and *ompF *displayed a significant change in their levels of expression being induced in the *crp*^- ^mutant and repressed in the presence of glucose. It has not been reported that CRP directly regulates these well characterized genes. Instead, CRP directly controls the expression of the *ompR *gene, whose product controls the expression of *ompF*. Our result is consistent with a report showing an increment in the expression level of *ompF *under glucose limitation [45]. The effect is caused by the absence of cAMP that increases the levels of phosphorylated OmpR, which repress expression of *ompF*.

We have presented some of the relevant observations that can be extracted from table 1 and the cluster analysis comparing the wild type and the *crp*^- ^mutant. This analysis has shown that, as has been pointed out before, catabolic repression is mainly controlled by CRP, but that a small set of genes respond as a consequence of the intervention of TFs that have no described relationship with CRP. On the other hand, the prevalent role of Fis in the activation of genes under the LB+G conditions becomes evident in this analysis. It is known that *fis *gene transcription levels respond to growth rate, as can be expected since cells in LB+G medium grow 5% faster than cells in LB. Interestingly, it was also found that in the *crp*^- ^mutant, a strain that grows 5% slower than the wild type strain in the same LB medium, *fis *transcript levels are increased 3 fold (Table 1). Thus, these results show that CRP is playing an important role in *fis *regulation, resulting in its derepression when glucose is present.

### Topological analysis of the regulatory network involved in the glucose response

The experimental results revealed that transcription factors CRP and Fis, are major regulators causing an extended response to glucose. However, it is clear that other TFs are also involved in controlling the genes found to respond to glucose. To help in identifying the relative roles of these TFs, an analysis of the properties of the regulatory network and its subnetworks (modules) is required. Resendis *et al *[28], demonstrate that the analysis of the regulatory network in terms of its topology will evidence the relationship between modules and physiological functional classes [28]. Starting from the identified RN, we then performed a topological analysis to identify modules involved in the observed transcriptional responses.

**Figure 3:**
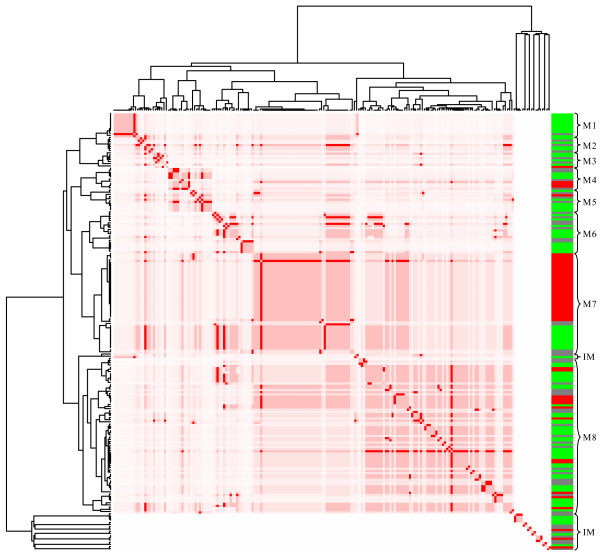
**Representation of the regulatory network as topological modules**. The figure illustrates the presence of several isolated modules and eight interconnected modules. The relative transcript levels of genes integrating the RN are shown in the right edge. Increased and decreased transcript levels are indicated by red and green, respectively.

**Figure 4:**
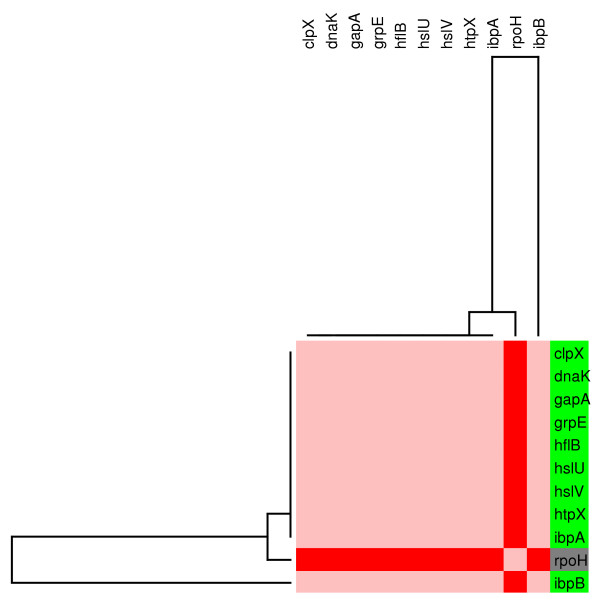
**Individual modular component of the regulatory network under the control of Sigma 32 (M1)**. The figure represents a detailed view of the individual topological module extracted from figure 3. The relative transcript levels and names of genes integrating the subnetwork are shown on the right. Decreased transcript levels are indicated in green. Grey indicates transcription factors that did not respond to glucose in the transcriptome experiment.

**Figure 12:**
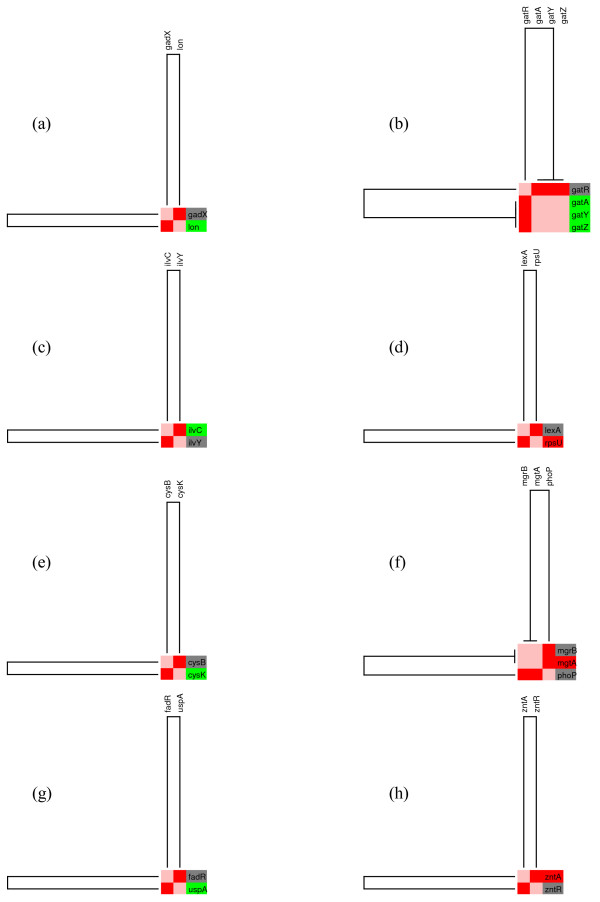
**Eight mini-modules disconnected from the giant component**. **(IM). **We illustrated a series of subnetworks that do not show any connection with the giant component in figure 3. The expression levels and color remains as previously described.

Our study revealed sets of genes grouped into one independent unit (figure 12) and eight connected topological modules (figure 3). Module 1 includes genes regulated primarily by the sigma factor RpoH (sigma32), related to the heat shock response and chaperone proteins. The levels of the HSPs are tightly coupled to the metabolic and environmental status of the cell by regulation at the transcriptional level. Homogeneous patterns of gene expression were observed for 10 affected genes that were repressed in the presence of glucose (figure 4). Even though no direct effect of CRP has been reported for these genes, it has been reported that the active form of CRP directly stimulates the expression of sigma32 [46]. This result is consistent with the observed repression of this group of genes that can be explained by inactive CRP in the presence of glucose or in a *crp*^- ^background.

**Figure 5:**
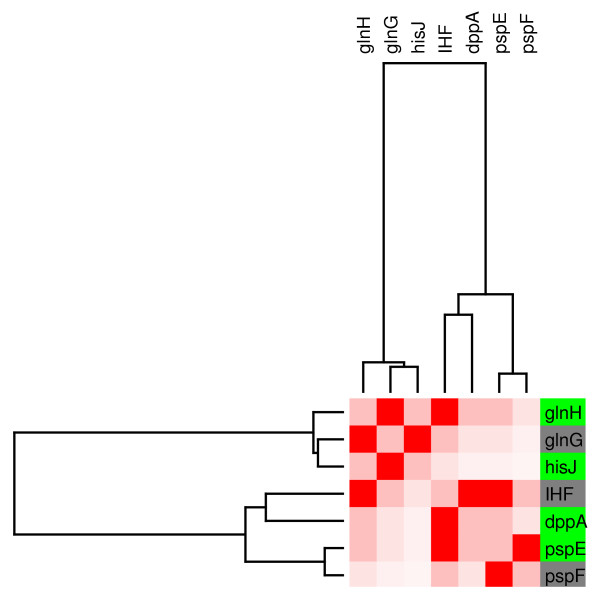
**Modular component of the regulatory network controled by IHF**. **(M2).**. Module 2, shows a zoom in to a subnetwork, that clustered a set of genes mainly regulated by IHF. As in figure 4, the relative transcript levels and names of genes integrating the subnetwork are shown on the right. Decreased transcript levels are indicated in green. Grey indicates transcription factors that did not respond to glucose in the transcriptome experiment.

Module 2 is controlled by Integration Host Factor (IHF). It includes 4 genes that are homogenously expressed, and are mainly related with amino acids metabolism and laterally transfered elements (figure 5).

**Figure 6:**
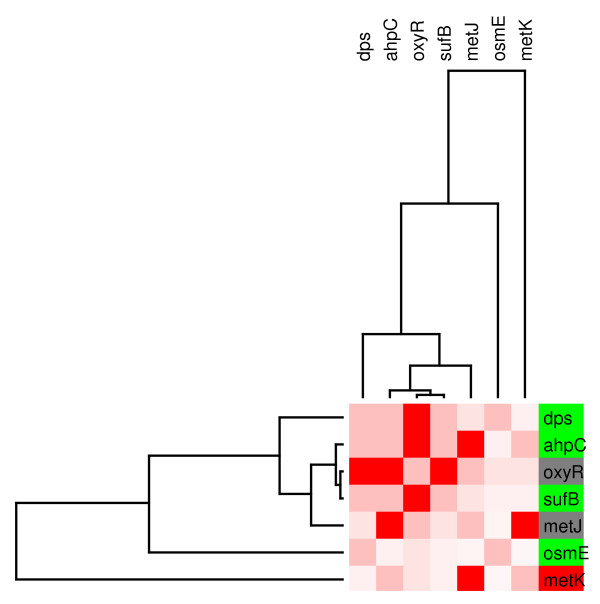
**The OxyR modular component of the regulatory network**. **(M3). **The figure illustrates the genes in the module that were up regulated (in red) and down regulated (in green). Grey indicates transcription factors that did not respond to glucose in the transcriptome experiment.

Module 3 is composed of five genes mainly regulated by the oxidative stress protein OxyR with some subnetworks also regulated by methionine repressor MetJ or IHF. We can observe in figure 6 that the genes corregulated by OxyR are homogeneously repressed. We also observed that all of the proteins corregulating this set except MetJ, function as activators. The gene *metK*, which appeared to be solely regulated by MetJ, was induced under this condition, suggesting that all of the regulators should be inactive in the presence of glucose.

**Figure 7:**
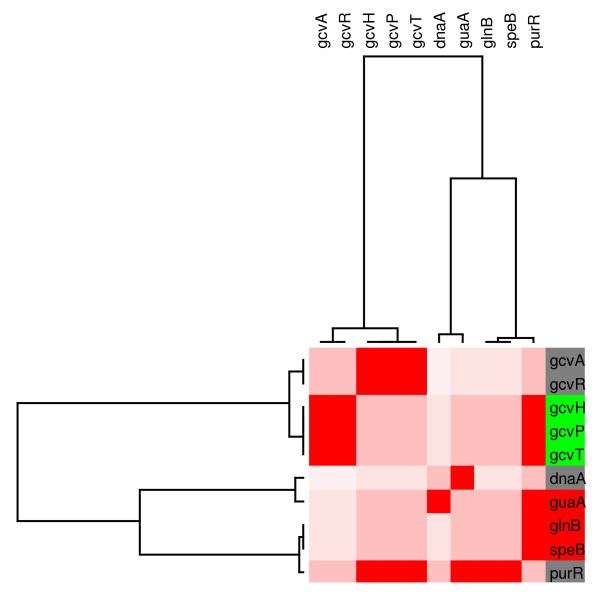
**The PurR modular component**. **(M4). **The figure shows the relative transcript levels and names of genes integrating the subnetwork at the right. Increased and decreased transcript levels are indicated by red and green, respectively. Grey indicates transcription factors that did not respond to glucose in the transcriptome experiment.

Module 4 (figure 7), is mainly composed of the PurR regulon that controls expression of the *gcvTHP *operon that is involved in glycine metabolism. These genes were down regulated in the presence of glucose, a phenomenon that has been studied by Wonderling and Stauffer [47]. The authors demonstrate that *crp *inactivation caused a reduction in *gcvT *expression in the presence of glucose. The other three genes (*guaA*, *glnB*, *spe*), appeared induced under this condition. In Table 1, the genes *glnB *and *speB *are exclusively regulated by PurR, acting as a repressor. If no other TF or alternative regulatory processes are affecting these genes, it would be possible to predict that the state of PurR is in *off *in the presence of glucose, therefore, it is not repressing transcription.

**Figure 8:**
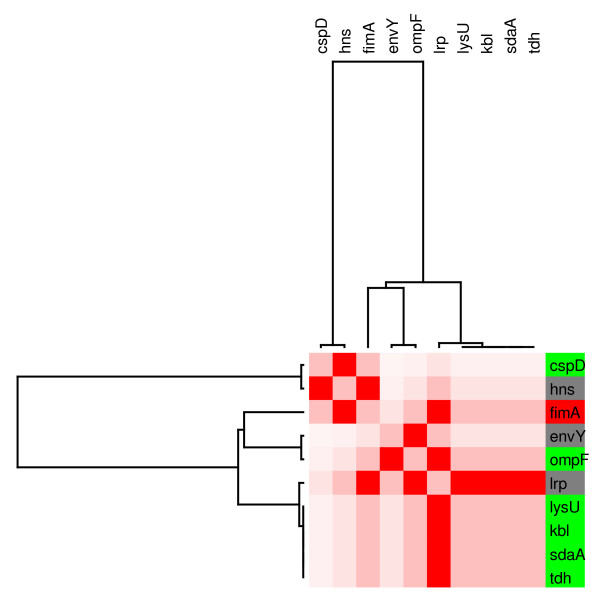
**Individual modular component of the regulatory network controled by Lrp**. **(M5). **The relative transcript levels and names of genes integrating the subnetwork mainly regulated by Lrp, are shown on the right. Six genes in the figure decreased their relative transcript levels (in green), and only one gene was induced in this module (in red). Grey indicates transcription factors that did not respond to glucose in the transcriptome experiment.

Module 5 is largely regulated by leucine-responsive regulatory protein Lrp (figure 8). Genes *kbl *and *tdh *belong to the same operon, according to the data extracted from RegulonDB. Lrp represses the expression of the operon. These two genes appeared down-regulated in the presence of glucose suggesting that Lrp is repressing their expression. The *ompF *and *fimA *genes exhibit a very complex regulation. EnvY and Lrp that act as activators, and IHF and OmpR that function as dual regulators of the *ompF *gene, are repressed under glucose conditions. A search of the literature revealed that our results are consistent with previous data that report that the expression of *ompF *is increased more than 40-fold higher under glucose limitation conditions [45]. In the same work, the authors reported expression of *ompF *in the absence of cAMP. The induced OmpR resulted in the production of more OmpR-P, which represses the expression of *ompF *gene.

**Figure 9:**
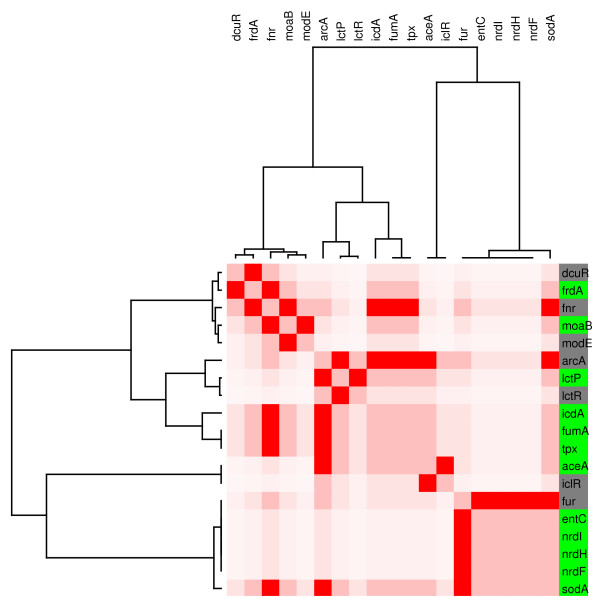
**Respiration module**. **(M6). **In this case, the module is controlled by a set of proteins kwon to be related to different respiration forms in E. coli. The expression levels and color remains as previously described.

**Figure 10:**
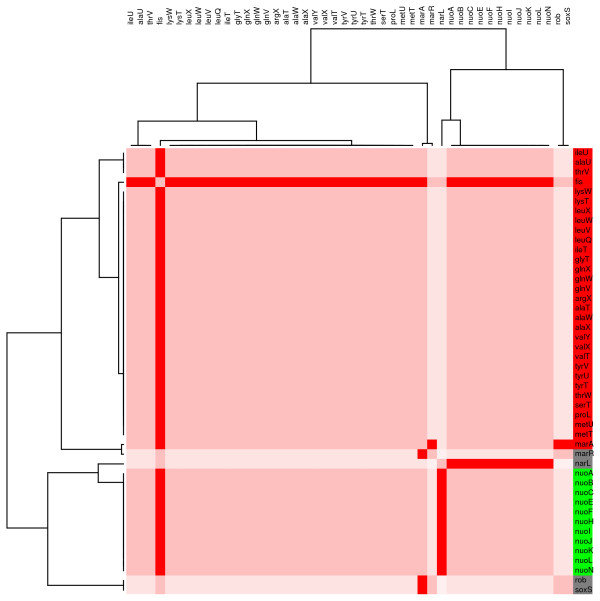
**Individual modular component of the regulatory network controlled by Fis**. **(M7). **A modular component mainly regulated by the transcriptional factor Fis. Increased and decreased transcript levels are indicated by red and green, respectively. Grey indicates transcription factors that did not respond to glucose in the transcriptome experiment.

Module 6 is composed of 12 genes related to respiratory or energy generation functions (figure 9). It was amazing to find that all the genes that constitute this module are homogenously expressed considering that, as for the CRP module, the genes are regulated by several factors controlling expression of these genes. Module 7 has 41 genes, regulated by Fis. Within subdivisions observed in the tree for these sub-branches, homogeneous gene expression patterns are observed that correspond to genes corregulated by the same set of regulatory proteins (figure 10).

**Figure 11:**
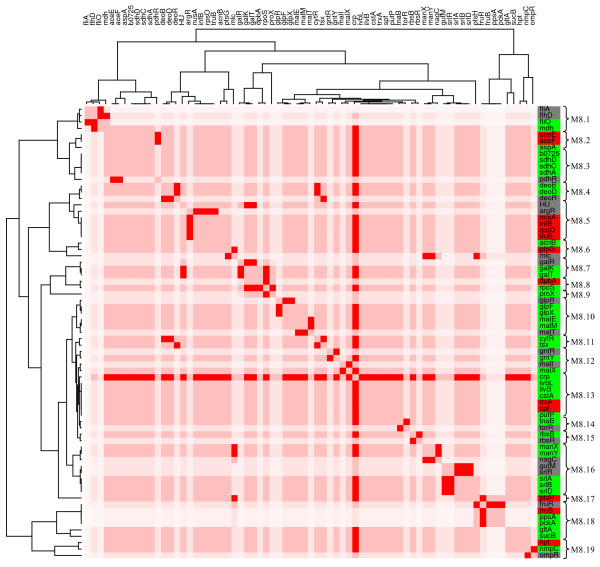
**Carbon sources assimilation module**. **(M8). **The figure ilustrates a module in which most of the genes are interconted by the CRP transcription factor. It is also divided in 19 submodules, that are related with other Tf's that corregulate with CRP. The expression levels and color remains as previously described.

Module 8 includes genes involved in carbon source assimilation. As mentioned before, the 50 genes of the CRP module do not present homogeneous gene expression patterns. However, by searching lower branches of the tree (figure 3 or table 1), we found that except for 2 sub-branches, the rest correspond to groups of genes corregulated by more than one TF. Following this criterion, we located 19 sub-branches (figure 11) in which 6 subgroups show non-homogeneous gene expression patterns. It is interesting to note that two of them are exclusively regulated by only one TF, CRP in the first case, and the fructose regulator FruR in the second. The differences in gene expression are given because the clustering algorithm does not consider the fact that the proteins can exert opposite effects on regulated genes, positive or negative. Interestingly, genes positively regulated appear closer to each other in the cluster than for negatively regulated genes. The other groups cluster genes corregulated by CRP and particularly accessory TFs.

We found four cases (submodules 8.2, 8.6, 8.8 and 8.19), in which one gene presents the opposite expression pattern compared to the other members of the group. In all cases, the gene with opposite expression pattern lacks one of the TF binding sites present in the other genes. An example is the subbranch containing the genes *aceE*, *aceF *and *aspA*, in which the first two genes are corregulated positively by CRP, positively or negatively by FNR, and negatively by PdhR. If we consider only the information found in RegulonDB and EcoCyc, the increased levels of expression of *aceE *and *aceF *should be a consequence of the inactivation of CRP and PdhR. Considering the low levels of cAMP, and the increase of pyruvate as a product of glycolysis [48], we can assume that FNR might activate or not repress the *aceE *and *aceF *genes. The *aspA *gene, which is positively regulated by CRP and FNR, appears down regulated in the presence of glucose. This result is consistent with the finding that *aspA *is under catabolite repression control [49,50].

**Figure 13:**
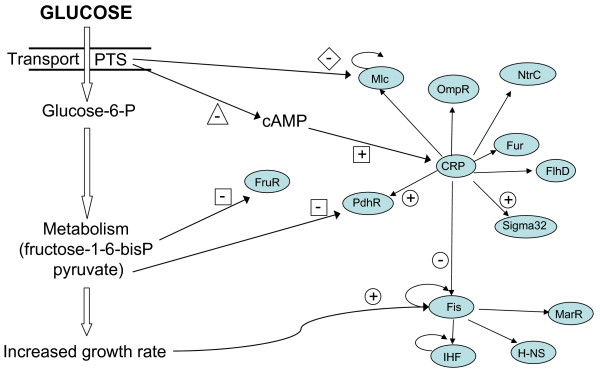
**Glucose sensing pathways and the TFs involved**. Plus and minus signs indicate positive and negative effects: (○) transcriptional control, (□) metabolites interaction, (◇) protein-protein interaction, and (△) adenylate cyclase control.

The preceding analysis provides a view of the roles and interactions of specific TFs in response to glucose. An important question related to this subject is: how many different pathways/mechanisms exist in *E. coli *to detect the presence of glucose and relay this information to the RN? Figure 13 was generated mainly from previously published works and it is supported by some of our current results. This figure shows a summary of the signals generated by the consumption of glucose and their effects on specific TFs. Specific glucose detection is dependent on the phosphorylation state of the glucose-specific PTS protein IIB^Glc^. This protein is involved in the phosphorylation of glucose that is transported by the IIC^Glc ^integral membrane protein domain. When glucose is present in the medium, IIB^Glc ^is mainly in a non-phosphorylated state. Under this condition, IIB^GC ^binds the Mlc repressor protein, thus relieving its repression of the *ptsHI *and *ptsG *genes, among others[16]. Other signals generated by the presence of glucose, such as a relatively low level of cAMP, increased levels of certain metabolites, and an increased growth rate are caused directly or indirectly. A clear effect of this phenomenon can be seen in figure 13 with fructose-1-6-biphosphate and pyruvate that induce the expression of genes under FruR and PdhR control. Sugars other than glucose can also cause some of these effects, but these will vary depending on their quality as carbon and energy sources. All these signals are detected by specific TFs that in turn regulate other TFs or structural genes. As shown in figure 13, some TFs can simultaneously receive and thus integrate inputs from different pathways, such as is the case with Fis, which displays growth-rate regulation and is also regulated by Crp. It should be emphasized, though, that we are still far from a complete understanding of how the glucose signal is propagated through this network, and how other environmental signals are integrated to modulate the overall response. The combined analyses of transcriptome data and the RN involved in the observed responses, as has been performed here, should contribute to the identification of signaling pathways and their integration by the RN.

## Conclusion

The analysis of transcriptome data collected under conditions of glucose deficiency and sufficiency in a complex medium enabled us to identify functions involved in the adaptation of *E. coli *to these two different growth conditions. The known repressive effects of glucose on gluconeogenesis and on alternative carbon source import and metabolism were clearly demonstrated. Furthermore, when glucose was present in the medium, an increase in overall protein synthesis capacity was observed. Also, responsive to the presence of glucose were genes encoding different cellular functions including cell division, replication, transcription, and the biosynthesis of cofactors, nucleic acids, amino acids and lipids. This analysis also revealed that functions related to proteolysis and protein folding are apparently more important when *E. coli *is growing in LB medium as compared with LB+G medium.

The topological analysis of the RN involved in the regulation of a subset of glucose-responsive genes, revealed eight modules including 37 TFs. Most of the RN topological modules include genes encoding functions with similar physiological roles, and together they represent a significant part of the glucose stimulon. The modules we identified partially correspond to the regulatory subnetworks originating at sensor TFs (origons) that have been identified in the complete *E. coli *RN[29]. The difference can be explained considering that we have limited our analyses to specific growth conditions and a subset of the RN. It can be assumed that this is still a partial representation of the RN involved in this response, since the functions of a significant number of TFs in *E. coli *are still unknown [30,51]. In spite of this shortcoming, our results and those previously reported by other groups indicate that CRP and Fis play a dominant role in the transcriptional responses detected in this study. This analysis places CRP and Fis as central TFs in the subset of the *E. coli *RN that senses and responds to glucose and other sugars. These two regulatory proteins integrate different types of signals that reflect the nutritional composition of the medium and the physiological state of the cell, causing a corresponding genome-wide transcriptional response.

Current limits in sensitivity and specificity for transcriptome analysis methodologies, together with our incomplete knowledge of the properties and interactions of TFs, still do not allow a thorough understanding of the cellular response to specific stimuli. However, integrative analysis of transcriptome and RN data as performed here, should provide a framework for the future generation of models representing the cell's capacity to respond to a changing environment.

## Methods

### Source of experimental data

Transcriptome data was obtained from previously reported experiments performed with *E. coli *strain BW25113 and an isogenic *crp *mutant (LJ3017)[10]. Briefly, strains were grown at 37°C with agitation in Luria-Bertani (LB) broth containing 50 mM potassium phosphate, pH 7.4, and 0.2 mM Lcysteine with or without 0.4% glucose. Cells were grown in triplicate in 25 ml of medium in shake flasks starting at an OD_600 _of 0.05 and harvested in the exponential growth phase when cultures reached an OD_600 _of 0.5. When grown in LB medium, generation times for strains BW25113 and LJ3017 corresponded to 37 and 43 min., respectively. In LB+G medium, generation times for strains BW25113 and LJ3017 corresponded to 35 and 41 min., respectively[10,21]. Total RNA was extracted from each sample, processed and hybrydized to the Affymetrix *E. coli *array which includes 4327 genes and intergenic regions[11].

### Data analysis

Array scanning, data collection and normalization were performed following the procedure described by Caldwell et al. 2001[52]. Three data sets were obtained for each of three experimental conditions: wild type grown in LB medium (WT), wild type grown in LB medium + glucose (WTg) and a *crp*^- ^mutant grown in LB medium (CRP). The data sets for each strain and condition were compared pair-wise to determine the Pearson correlation coefficient. For each triplicate data set, the two sets with the highest Pearson correlation coefficient were retained for further analysis.

For each pair of data sets of all experimental conditions, the reliability of the data for each gene was calculated according to the Affymetrix statistical algorithms reference guide (Affimetrix, Inc., 2004). A "Present" absolute call is assigned to a gene when the signal/noise ratio is higher than an internally calculated threshold. When signal value data for each gene displayed a "Present" absolute call in both duplicate experiments, both values were considered to be reliable. The two signal values for that gene were averaged, and the resulting data were used in subsequent analyses. Using this approach, the number of genes considered for further analysis corresponded to 1908, 1910 and 3083 for WT, WTg and CRP conditions, respectively. Using the signal averages for each condition, we then calculated the WTg/WT and CRP/WT log ratios.

### Identification of differentially transcribed genes

Differentially transcribed genes were selected using an outlier iteration method [12-14]. The method consists in calculating the average and the standard deviation (SD) of the log ratio for all sets of genes under the four conditions. In order to identify significant levels of gene expression, we assumed that the threshold value of significance is two SD. Thus, any gene with a log ratio higher than two SD from the mean is considered an outlier. Outliers were removed from the population and gathered in a differentially expressed subset. For the rest of the genes, we calculated again the averages of their log ratios and their SD values. Selection of the outliers was determined as in the previous case. The process was repeated until no outliers were detected in each situation. Using this method, the number of genes selected corresponded to 380 for WTg/WT and 333 for CRP/WT. For CRP/WT, 196 genes were down regulated and 137 up regulated. Table S1 shows the genes identified in this study, where values for WTg/WT and CRP/WT log ratios are provided. In addition, when known, the regulatory phrase for each gene is indicated and also, when a gene is part of an operon, the genes belonging to it are indicated. Information about gene functions and operon organization was obtained from RegulonDB [53] and EcoCyc [54]. It should be pointed out that the terms "induced" and "repressed" are used in this work to indicate increased or decreased transcript levels, respectively. These terms do not imply a particular mechanism of gene regulation.

### Clustering of microarray data

We applied a hierarchical centroid linkage clustering algorithm[41,55] with correlation uncentered as similarity measure, to the WTg/WT and CRP/WT log ratios. The clustering results were visualized using the Treeview program[56].

### Extraction of condition-specific subnetworks

For each microarray condition (WTg/WT or CRP/WT), we reconstructed a condition specific subnetwork as follows. From the transcriptional regulatory network (RN) of *E. coli*, we extracted the genes identified for each microarray condition, the TFs regulating their expression, and the transcriptional interactions between TFs and regularted genes. In these subnetworks, nodes represent genes, and edges represent the transcriptional interactions. Known regulatory sites and transcriptional unit organization were obtained from RegulonDB )[30] and EcoCyc[57].

### Identification of condition-specific modules

We identified the WTg/WT condition-specific modules applying to the condition specific subnetwork the methodology described in Resendis-Antonio et al[28]. That is to say, we clustered the genes based on their shortest distance within the network. Afterwards, we annotated each gene with its corresponding microarray expression level.

## Abbreviations

LB, LB+G WTg, WT, TF, RN, PTS.

## Authors' contributions

RMG contributed with the computer analysis and interpretation of the microarray data in terms of the regulatory network, elaboration of programs for data management and discussion for the selection and processing methods. JF contributed with computer analysis of microarray data and with the construction of topological modules. OR processed microarray data, evaluating different approaches for the identification of differentially transcribed genes. JCV participated in data anlaysis and the discussion of every section of the manuscript. MS particpated in the experimental design, supplying the microarray data and collaborating in the discussion of every section of the manuscript. GG participated in the experimental design and contributed with the analysis and interpretation of microarray data for every section of the manuscript.
